# Dietary low levels of *Eucommia ulmoides* leaf extracts: effects on antioxidant capacity, immunity, and cecal microbiota in lipopolysaccharide-challenged broilers

**DOI:** 10.3389/fmicb.2025.1662502

**Published:** 2025-10-03

**Authors:** Jinliang Zhang, Lei Chai, Yan Guo, Jincheng Han, Guangli Yang

**Affiliations:** ^1^College of Biology and Food, Shangqiu Normal University, Shangqiu, Henan, China; ^2^College of Smart Animal Husbandry, Shangqiu Normal University, Shangqiu, Henan, China; ^3^Jiaozuo Product Quality Inspection and Testing Center, Jiaozuo, Henan, China

**Keywords:** broiler, *Eucommia ulmoides* leaf extract, antioxidant capacity, immunity, cecal microbiota

## Abstract

**Background:**

Environmental stimuli exerts detrimental effects on health and production performance during intensive animal production with ensuing serious economic consequences. This study aimed to investigate the effects of dietary low levels of *Eucommia ulmoides* leaf extracts (EULE) on antioxidant capacity, immunity, and cecal microbiota in lipopolysaccharide (LPS) -challenged broilers.

**Methods and Results:**

In this study, a total of 400 21-day-old Ross 308 male broilers were divided into 5 groups, with 8 replicates per group and 10 birds per replicate. They were fed a basal diet (CON group and LPS group) or a basal diet supplemented with 100, 200, or 300 mg/kg EULE (EULE100, EULE200, and EULE300 groups) for 10 consecutive days. The results showed that growth performance and relative organ weights were not affected by either LPS injection or EULE supplementation (*p <* 0.05), but dietary supplementation with EULE decreased the increased relative organ weights in LPS-induced broilers. LPS challenge decreased the level of catalase (CAT), *Faecalibacterium*, and increased the level of Interleukin 4 (IL-4), which were improved by EULE (*p <* 0.05). Furthermore, EULE200 and EULE300 reduced the levels of IL-1β, IL-2 and IL-6 in LPS-induced broilers. EULE300 significantly decreased the serum malondialdehyde (MDA) level and increased the level of total antioxidant capacity (T-AOC), and reduced the proportion of harmful genera *Erysipelatoclostridium* in cecum.

**Conclusion:**

This study emphasizes that dietary inclusion EULE, even low levels (100, 200, or 300 mg/kg), can exhibit significant anti-inflammatory and anti-oxidation effects, modulate cecal microbiota structure and restore cecum barrier function caused by LPS stimulation.

## 1 Introduction

In the livestock industry, broilers face multiple stressors during growth. Some stress factors can induce immune stress, such as improper temperature and humidity, poor air conditions, vaccination, and microbial infection, etc., which will decrease the immune function of the body, reduce the production performance and even increase the mortality (Tiku and Tan, 2021; Hu et al., 2024). The harm of immune stress to animal production has become more and more serious, and it has become an important problem to solve the harm of immune stress to animal breeding industry. In recent years, plant extracts have been proved to have many effects, such as enhancing immunity, anti-oxidation, antitumor, anti-aging, etc., and have been widely used as functional nutritional factors to maintain animal health and improve animal performance (Loi et al., 2020). And numerous studies have demonstrated that plant extracts can have a positive impact on the growth performance and immune function (Müştak et al., 2015; Ji et al., 2024; Liu et al., 2024). Lipopolysaccharide (LPS), also known as endotoxins, a primary component of the cell wall of gram-negative bacteria such as *Escherichia coli* and *Salmonella*, can be recognized by immune cells as a pathogen-associated molecular pattern and consequently induces an inflammatory response (Shi et al., 2022; Wang X. et al., 2024; Li et al., 2025). Intraperitoneal or intravenous injection of LPS can be used to effectively model oxidative stress and inflammatory damage caused by bacterial infection (Shi et al., 2022). Therefore, LPS is often used to establish immune stress models in various animals, such as broiler (Xia et al., 2025), pig (Wang Q. et al., 2024), and mice (Zhou et al., 2021). Studies have reported that nutritional interventions [such as probiotics (Ding et al., 2024), polysaccharide (Xing et al., 2023), and natural plant extract (Shi et al., 2022)] could effectively alleviate LPS-induced immune overresponse in broilers. However, whether the low level of EULE exerts beneficial effects on growth performance and immune function in broilers still remains unclear.

*Eucommia ulmoides* is a unique relic plant in China and was first recorded in the ‘Shennong’s Herbal Classic’ of the Han Dynasty, more than 2000 years ago. At present, *Eucommia ulmoides* has been officially used as a medicinal plant and included in the ‘Chinese harmacopoeia’. *Eucommia ulmoides* leaf extract (EULE) had been included in the variety catalogue of feed additives of China. The leaf extracts mainly include Eucommia flavone, Eucommia polysaccharides and Chlorogenic acid, respectively. Among them, Chlorogenic acid (CGA) is one of the main active components. CGA has antioxidant, anti-inflammatory, antibacterial, antiviral, anticancer and other physiological functions, has been widely used in livestock and poultry production. Studies have shown that CGA can improve animal performance (Bai et al., 2022; Song et al., 2024), intestinal environment (Liu et al., 2022; Li Y. et al., 2023; Tan et al., 2023) and enhanced immune function (Lv et al., 2023; Liu H. et al., 2023). In addition, CGA can improve the meat quality of oxidatively stressed broilers (Zhao et al., 2019; Zhang et al., 2022).

The entire plant of *Eucommia ulmoides* can be used as medicine. Among them, the leaves of *Eucommia ulmoides* are particularly valuable due to their large yield, abundant resources, and rich nutritional components, including vitamin B1, vitamin E, β-carotene, 17 kinds of free amino acids, and 15 kinds of trace elements such as germanium and selenium. Therefore, how to turn waste into treasure and maximize the utilization of resources has become the focus of people’s attention. Currently, studies have proved that EULE shows positive effects on the growth performance and health of broiler chickens. Therefore, in-depth exploration of the impact of EULE on broilers production and its mechanism is of great research value. In particular, the effect of low levels of EULE on health and growth performance in LPS-induced broilers. Therefore, this study aimed to evaluate the low dose of EULE and antioxidant performance of broilers by means of production performance, organ development, blood biochemical indexes, antioxidant performance and cecum microflora, and finally revealed the protective mechanism of EULE (CGA 98%) on LPS-challenged broilers. The results of the study provide guidance for the development and application of new feed additives and the ‘no-resistance’ nutrition program of broilers.

## 2 Materials and methods

The procedures used in this research were approved by the Animal Ethics Committee of Shangqiu Normal University (2021-1,025).

### 2.1 Animals, diets and experimental design

Four hundred, 21-day-old Ross 308 male broilers were weighed and divided into 5 groups with 8 replicates in each group: (1) CON group, injected with saline and fed the basal diet; (2) LPS group, injected with 1 mg lipopolysaccharide (LPS)/kg body weight (BW) and fed with the basal diet; (3) EULE100 group, injected with 1 mg LPS/kg BW and fed with the basal diet supplemented with 100 mg/kg EULE; (4) EULE200 group, injected with 1 mg LPS/kg BW and fed with the basal diet supplemented with 200 mg/kg EULE; and (5) EULE300 group, injected with 1 mg LPS/kg BW and fed with the basal diet supplemented with 300 mg/kg EULE. Feed was formulated according to recommendations of the National Research Council (National Research Council Subcommittee on Poultry Nutrition, 1994) and the composition of the basic diet was the same as for the previous experiment (Guo et al., 2023). The broilers were provided *ad libitum* feed and water. EULE (CGA 98%) was purchased from Changsha Staherb natural ingredients Co., (Changsha, China) and thoroughly mixed into the basal diet. LPS was purchased from Sigma Co. and the serotype of *E. coli* is O55: B5.

The broilers were reared in stainless steel cages with dimensions of 140 cm (length), 70 cm (width), and 35 cm (height). The experiment lasted until 31 days. During the experiment, all birds were kept at 24 ± 2 °C, and the relative humidity was 50–60%. Broilers were exposed to 24 h of continuous light. EULE was added throughout the experiment. Saline or LPS was injected intraperitoneally at the 28th day of age. At 72 h after the injection, one bird from each repeat was selected and euthanized.

### 2.2 Growth performance

Broilers were weighed on days 21 and 31 of age, which were used to calculate weight gain (WG) and feed consumption during the experiment was recorded to calculate average daily gain (ADG) and average daily feed intake (ADFI). Feed conversion ratio (FCR) was determined by dividing ADFI by ADG.

### 2.3 Sample collection

At the end of the experiment, samples were collected. Blood samples were collected from the wing vein, centrifuged for 10 min (3,000 rpm) at 4 °C, and the serum was separated and stored at −20 °C until analysis. After slaughter, bird heart, liver, spleen, lung, kidney, thymus, bursa and pancreas were isolated and weighed. Finally, the ceca contents were collected in sterile centrifuge tubes, flash frozen in liquid nitrogen, and stored at −80 °C for further analysis.

### 2.4 Relative organ weights

According to Ding et al. (2020), relative organ weight was calculated. Relative organ weight (g/kg) = organ weight (g)/live body weight (kg).

### 2.5 Analysis of serum parameters

Interleukin 2 (IL-2), IL-4, IL-6, and IL-1β levels were measured in serum using the enzyme-linked immunosorbent assay (ELISA) kits specific for poultry according to the manufacturer’s instructions (Jiancheng Biotechnology Co. Ltd., Nanjing, China). At the same time, the immunoglobulin A (IgA) and IgG levels in serum were determined with ELISA kits specific for chicken according to the manufacturer’s instructions (Shanghai Mlbio Co., Ltd. Shanghai China).

The oxidation-stress related indicators were determined as described by Guo et al. (2023). The level of T-AOC in bird serum was detected with ELISA kits specific for poultry (Jiancheng Biotechnology Co. Ltd., Nanjing, China). The CAT level was measured using the molybdate colorimetric method. The glutathione peroxidase (GSH-Px) level was detected using the 5,5′-dithio-nitrobenzoic acid (DTNB) chromogenic method. Superoxide dismutase (SOD) in serum was measured by the modified pyrogallol autoxidation method. MDA was measured by the modified thiobarbituric acid reactive substances assay.

### 2.6 Analysis of cecal microbiota

The intestinal contents were sent to in Shanghai Majorbio Bio-pharm Technology Co., Ltd. (Shanghai, China) for 16S rRNA gene sequencing. Total microbial DNA was extracted from ceca contents (100 mg) using the OMG-Soil, PF Mag-Bind Soil DNA Kit (Omega Bio-Tek, Georgi, USA). The V3-V4 region of the 16S rRNA gene was amplified by universal primers 338F: 5′-ACTCCTACGGGAGGTWTCTAAT-3′, and 806R: 5′-GGACTACHVGGGTWTCTAAT-3′. PCR products were obtained with a PCR Clean-Up Kit (Shanghai Meiji Yuhua Biomedical Technology Co., Ltd., Shanghai, China). Purified amplicons were pooled in equimolar concentrations and paired-end sequenced on an Illumina MiSeq PE300 platform/NovaSeq PE250 platform (Illumina, San Diego, CA, USA). Raw 16S rRNA sequencing reads were demultiplexed, quality-filtered, and merged as described by Ma et al. (2023).

High-quality reads were selected from raw sequencing data obtained on an Illumina platform, quality filtered, denoised, spliced and de-chimerized using the DADA2 method. Operational taxonomic units (OTUs) with a 97% similarity cutoff (Edgar, 2013) were clustered in UPARSE version 7.1 (Edgar, 2013), while chimeric sequences were removed. The taxonomy of each OTU representative sequence was analyzed in Ribosomal database project classifier version 2.2 (Wang et al., 2007) against the 16S rRNA Silva v138 database using a confidence threshold of 0.7. Bacterial diversity and linear discriminant analysis effect size were both performed using the online platform Majorbio Cloud Platform^1^ (Ren et al., 2022). To highlight the shared and distinct OTUs across the five groups, Venn diagrams were constructed by using R (version 3.1.1). Mothur (version 1.30.2) was employed to investigate the alpha diversity and rarefaction curve, and Qiime (version 1.9.1) were used to analysis the beta diversity and community barplot. The alpha diversity was revealed by Sobs, Ace, Chao1, Shannon, and Simpson indices, while beta diversity was utilized the principal components analysis (PCA) to demonstrate. The non-parametric ANOSIM test was employed to evaluate the differences between groups. The ribosomal database program classifier was applied to assign taxonomic levels down to the genus level, including the kingdom, phylum, class, order, and family. The microbiota with linear discriminant analysis (LDA) threshold >2.5 were identified using the linear discriminant analysis effect size (LEfSe) method.

### 2.7 Statistical analysis

All analyses were carried out using IBM-SPSS 26.0 software (SPSS. Inc., Chicago. IL). Data were analyzed using one-way ANOVA. Duncan’s multiple comparison was used to compare the differences among the five groups. The mean of 1 broiler per replicate served as an experimental unit for statistical analysis, a significance level of *p* < 0.05 was considered.

## 3 Results

### 3.1 Growth performance

The effects of EULE on growth performance in LPS-challenged broilers are shown in Table 1, there were no significant differences in ADFI, ADG and FCR among the five treatment groups (*p* > 0.05).

**Table 1:** Effects of *Eucommia ulmoides* leaf extracts (EULE) on growth performance of broilers.

Item	CON	LPS	EULE100	EULE200	EULE300	SEM	*p*-value		
							ANOVA	Linear	Quadratic
ADG (g)	45.95	41.83	45.99	46.43	44.08	1.405	0.453	0.553	0.807
ADFI (g)	70.96	66.32	71.60	71.00	68.93	1.928	0.924	0.871	0.667
FCR	1.54	1.59	1.56	1.52	1.56	0.072	0.085	0.215	0.158

CON, control group; LPS, challenged with LPS; EULE100, challenged with LPS and fed with the basal diet supplemented with 100mg/kg EULE; EULE200, challenged with LPS and fed with the basal diet supplemented with 200mg/kg EULE; EULE300, challenged with LPS and fed with the basal diet supplemented with 300mg/kg EULE. LPS, Lipopolysaccharide; EULE, *Eucommia ulmoides* leaf extracts.

No shoulder label in the same row indicates no significant difference (*P* > 0.05).

### 3.2 Relative organ weights

Although the relative organ weights were not affected by either LPS injection or EULE supplementation (*p* > 0.05) (Table 2), it was also found that EULE had a certain effect on the internal organs of LPS-challenged broilers. As shown in Table 2, compared to the CON, LPS stimulation increased the relative organ weights. Remarkably, dietary supplementation with EULE decreased the increased relative organ weights, in comparison to the LPS group. These findings suggested that the addition of EULE to the diet could both prevent and alleviate the negative effects of LPS stimulation on the vital organ tissue morphology of Ross 308 broilers.

**Table 2:** Effects of *Eucommia ulmoides* leaf extracts (EULE) on the relative organ weights of stressed broilers.

Relative weight (g/kg BW)	CON	LPS	EULE100	EULE200	EULE300	SEM	*p*-value		
							ANOVA	Linear	Quadratic
Heart	5.18	7.05	6.71	6.32	6.45	0.257	0.190	0.145	0.209
Liver	25.12	28.22	27.21	27.00	26.96	0.482	0.373	0.243	0.344
Spleen	0.72	3.19	0.87	0.95	1.00	0.311	0.057	0.803	0.076
Thymus	2.00	2.53	2.12	2.28	2.36	0.074	0.166	0.376	0.248
Bursa fabricius	2.41	2.73	2.49	2.91	2.57	0.116	0.676	0.235	0.853
Lung	5.61	6.32	6.38	5.45	6.14	0.208	0.534	0.838	0.097
Kidney	6.97	7.90	6.93	7.24	6.52	0.242	0.484	0.274	0.523
Pancreas	3.44	3.93	3.66	3.75	3.52	0.067	0.158	0.093	0.172

CON, control group; LPS, challenged with LPS; EULE100, challenged with LPS and fed with the basal diet supplemented with 100mg/kg EULE; EULE200, challenged with LPS and fed with the basal diet supplemented with 200mg/kg EULE; EULE300, challenged with LPS and fed with the basal diet supplemented with 300mg/kg EULE. LPS, Lipopolysaccharide; EULE, *Eucommia ulmoides* leaf extracts.

No shoulder label in the same row indicates no significant difference (*P* > 0.05).

### 3.3 Antioxidant capacity and immunity

**Figure 1:**
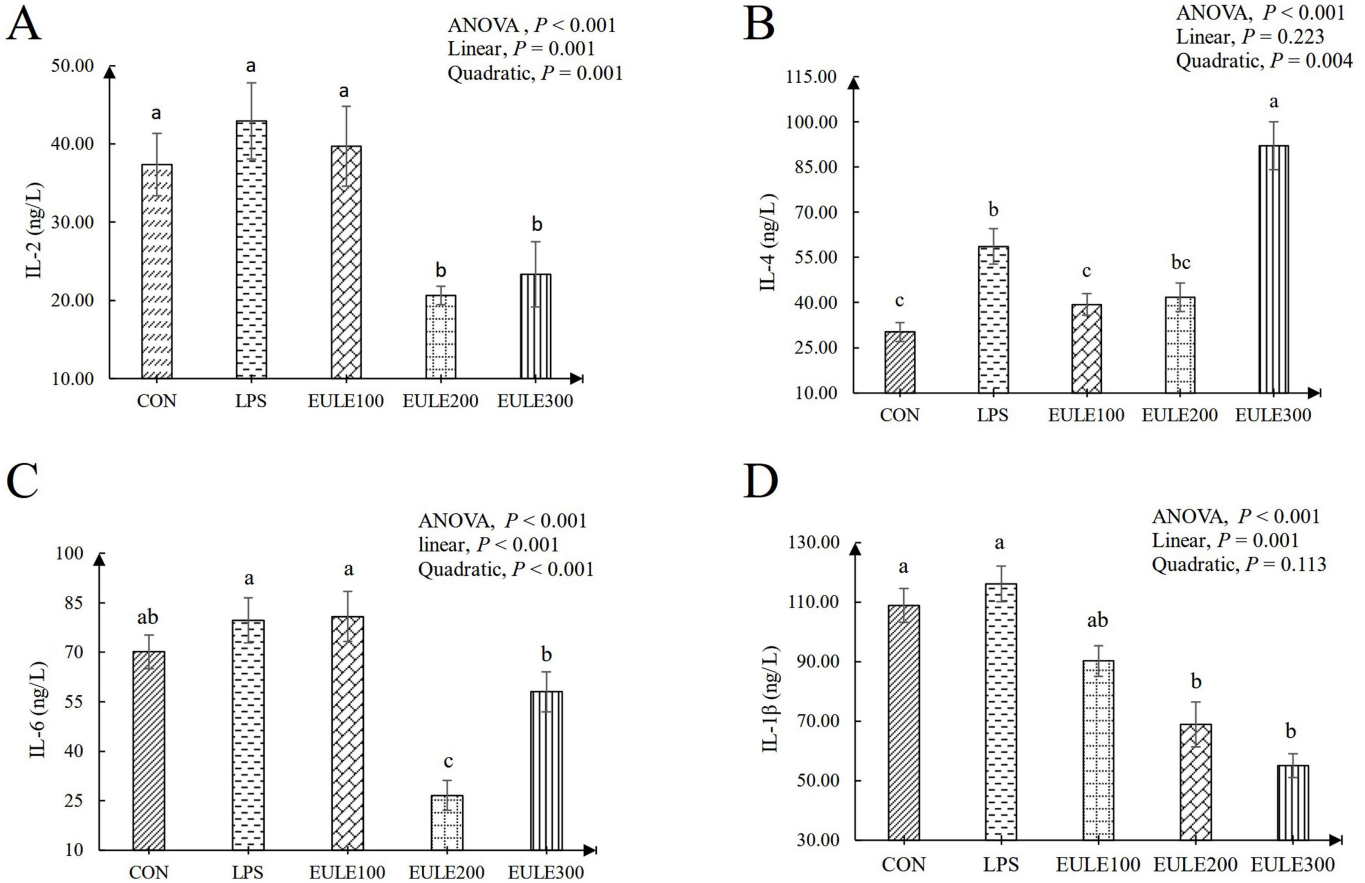
Effects of *Eucommia ulmoides* leaf extracts (EULE) on serum cytokine levels of broilers challenged with LPS. **(A)** IL-2 levels of broilers challenged with LPS. **(B)** IL-4 levels of broilers challenged with LPS. **(C)** IL-6 levels of broilers challenged with LPS. **(D)** IL-1β levels of broilers challenged with LPS. IL, Interleukin; LPS, Lipopolysaccharide, ^a,b,c^Different letters above the bars denotes a significantly different among groups.

**Figure 2:**
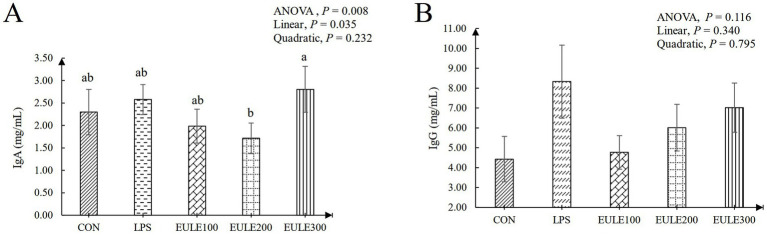
Effects of *Eucommia ulmoides* leaf extracts (EULE) on serum immunoglobulin levels of broilers challenged with LPS. **(A)** IgA levels of broilers challenged with LPS. **(B)** IgG levels of broilers challenged with LPS. Ig, Immunoglobulin; LPS, Lipopolysaccharide, ^a,b,c^Different letters above the bars denotes a significantly different among groups.

As shown in Table 3, compared to the CON group, significantly decreased levels of CAT were observed in the LPS group and the EULE supplementation groups (*p* < 0.05). Serum SOD and GSH-Px levels were not affected by either LPS stimulation or EULE supplementation (*p* > 0.05). EULE300 significantly decreased the serum MDA level and increased the level of T-AOC compared to the LPS group (*p* < 0.05). As illustrated in Figure 1, although the values of IL-2, IL-6, and IL-1β in the LPS group were higher than those in the CON group, which were not affected by LPS stimulation (*p* > 0.05). However, the EULE200 and EULE300 had lower serum IL-2, IL-4 and IL-1β levels in contrast to the other three groups (*p* < 0.05). It was further found that the IL-4 level in the LPS group was increased (*p* < 0.05) compared with the CON group (Figure 1B). Notably, EULE100 significantly decreased the IL-4 level (*p* < 0.05), while EULE300 significantly increased the IL-4 level (*p* < 0.05) (Figure 1B). As shown in Figure 2A, EULE200 decreased the IgA level, while EULE300 increased the IgA level (*p* < 0.05). IgG level was not affected by either LPS injection or EULE supplementation (*p* > 0.05) (Figure 2B).

**Table 3:** Effects of *Eucommia ulmoides* leaf extracts (EULE) on antioxidant indices in the serum of broilers.

Item (U·mg^−1^)	CON	LPS	EULE100	EULE200	EULE300	SEM	*p*-value		
							ANOVA	Linear	Quadratic
CAT	21.42^a^	5.39^b^	8.66^b^	10.15^b^	10.86^b^	1.434	0.001	< 0.001	0.090
SOD	220.51	143.52	162.43	174.82	216.75	15.954	0.491	0.981	0.180
GSH-Px	605.30	402.40	460.30	494.70	647.70	49.206	0.632	0.738	0.053
MDA	4.02^ab^	7.43^a^	5.28^ab^	4.28^ab^	1.57^b^	0.593	0.020	0.085	0.019
T-AOC	17.27^b^	3.45^b^	8.39^b^	9.50^b^	67.22^a^	5.564	<0.001	<0.001	<0.001

CON, control group; LPS, challenged with LPS; EULE100, challenged with LPS and fed with the basal diet supplemented with 100 mg/kg EULE; EULE200, challenged with LPS and fed with the basal diet supplemented with 200 mg/kg EULE; EULE300, challenged with LPS and fed with the basal diet supplemented with 300 mg/kg EULE. LPS, Lipopolysaccharide; EULE, *Eucommia ulmoides* leaf extracts.

^a,b^Values with different superscripts in the same row are significantly different (*p* < 0.05), no shoulder label in the same row indicates no significant difference (*p* > 0.05).

### 3.4 Composition and diversity of ceca microbial Flora

**Figure 3:**
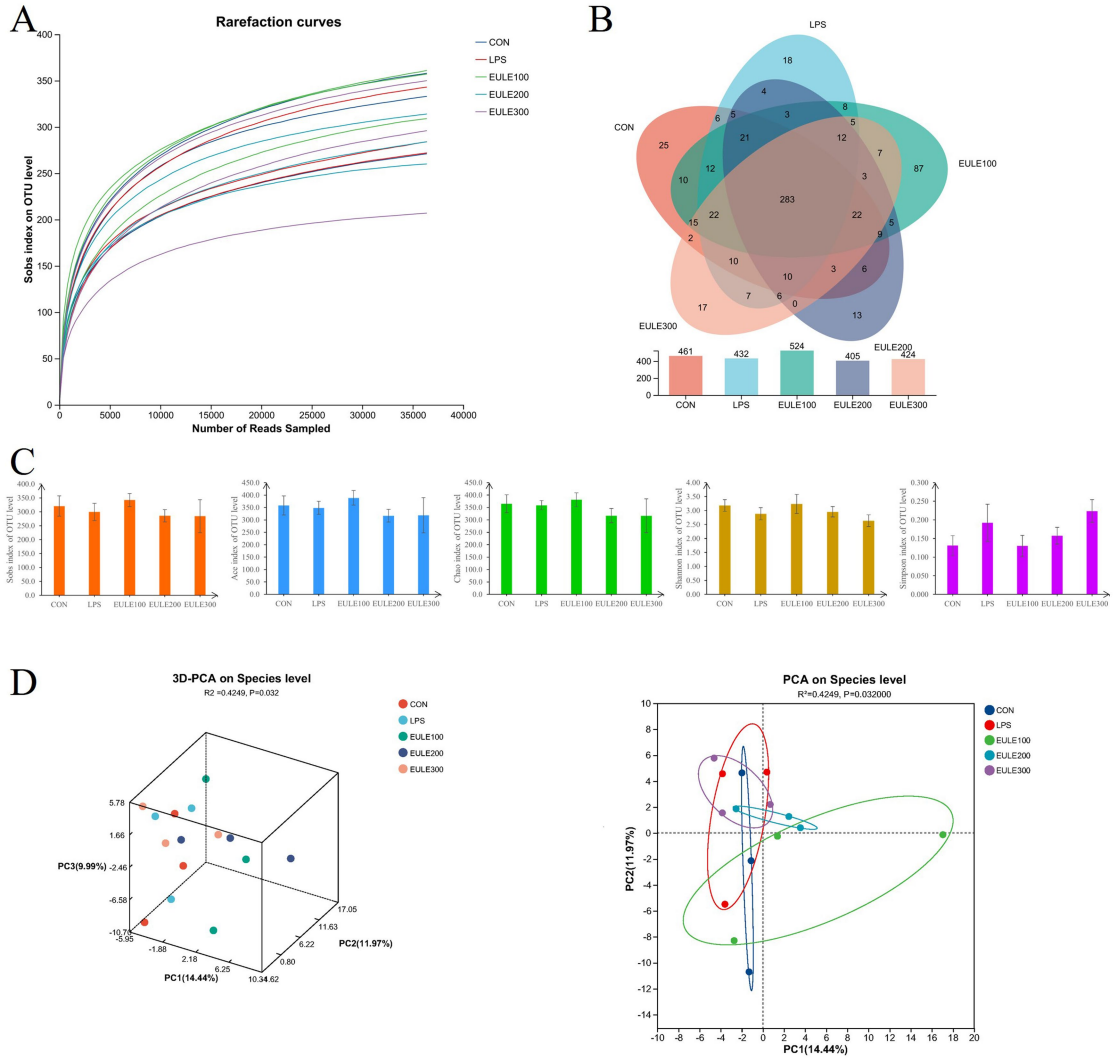
Effects of *Eucommia ulmoides* leaf extracts (EULE) on cecal microbiota diversity in LPS-challenged broilers. **(A)** Microbial rarefaction curves based on OTU level were used to assess the depth of coverage for each sample. Each treatment samples were distinguished by different colors of lines. **(B)** Venn diagram of OTUs level. **(C)** The alpha diversity paraments including Sobs, Ace, Chao, Shannon, and Simpson index. **(D)** Principal component analysis (PCA) scores plot of the samples.

As shown in Figure 3, clustering of microbes in the ceca microbiota was observed among the five groups. An average of 36,407 clean reads obtained from each ceca sample. The sequence number was more than 30,000 reads, while the OUT number kept unchanged (Figure 3A), indicated that the sequencing depth was sufficient to adequately reflect the microbial community composition of the ceca samples. Venn diagrams (Figure 3B) shown that 283 OTUs were shared among the five treatment groups, while the CON, LPS, EULE100, EULE200, and EULE300 groups were found to contain 461, 432, 524, 405, and 424 distinct OTUs, respectively. And as shown in Figure 3B, 25, 18, 87, 13, and 17 unique OTUs were identified in the CON, LPS, EULE100, EULE200, and EULE300 groups, respectively. As shown in Figure 3C; Table 4, the alpha diversity parameters (including Sobs, Ace, Chao, Shannon, and Simpson index) were not different among the five treatment groups (*p* > 0.05). R value calculated by ANOSIM was above 0, indicating there were greater difference between groups. The principal components analysis (PCA) (Figure 3D) showed that there was greater difference between groups (*p* < 0.05), and EULE dietary had distinguishable clustering with the LPS group while the principal component axes PC1, PC2, and PC3 explained 14.44, 11.97, and 9.99% of the total variation, respectively.

**Table 4:** Effects of *Eucommia ulmoides* leaf extracts (EULE) on cecal microbiota alpha diversity paraments in LPS-challenged broilers (OTU level).

Group	Richness index			Diversity index		Coverage/%
	Sobs	Ace	Chao	Shannon	Simpson	
CON	320.7 ± 44.79	358.3 ± 46.70	365.0 ± 44.14	3.179 ± 0.2570	0.1310 ± 0.03296	0.9985 ± 0.0001609
LPS	299.7 ± 38.00	348.6 ± 32.13	358.9 ± 22.53	2.883 ± 0.2674	0.1920 ± 0.06135	0.9984 ± 0.0001264
EULE100	342.3 ± 28.94	388.9 ± 36.25	380.9 ± 34.28	3.235 ± 0.4157	0.1300 ± 0.03496	0.9984 ± 0.0001922
EULE200	286.0 ± 27.06	316.8 ± 31.33	316.9 ± 35.11	2.955 ± 0.2281	0.1572 ± 0.02792	0.9988 ± 0.0003161
EULE300	284.3 ± 72.21	319.1 ± 86.91	316.2 ± 83.76	2.638 ± 0.2561	0.2239 ± 0.03714	0.9987 ± 0.0004681

CON, control group; LPS, challenged with LPS; EULE100, challenged with LPS and fed with the basal diet supplemented with 100 mg/kg EULE; EULE200, challenged with LPS and fed with the basal diet supplemented with 200 mg/kg EULE; EULE300, challenged with LPS and fed with the basal diet supplemented with 300 mg/kg EULE. LPS, Lipopolysaccharide; EULE, *Eucommia ulmoides* leaf extracts.

No shoulder label in the same row indicates no significant difference (*P* > 0.05).

**Figure 4:**
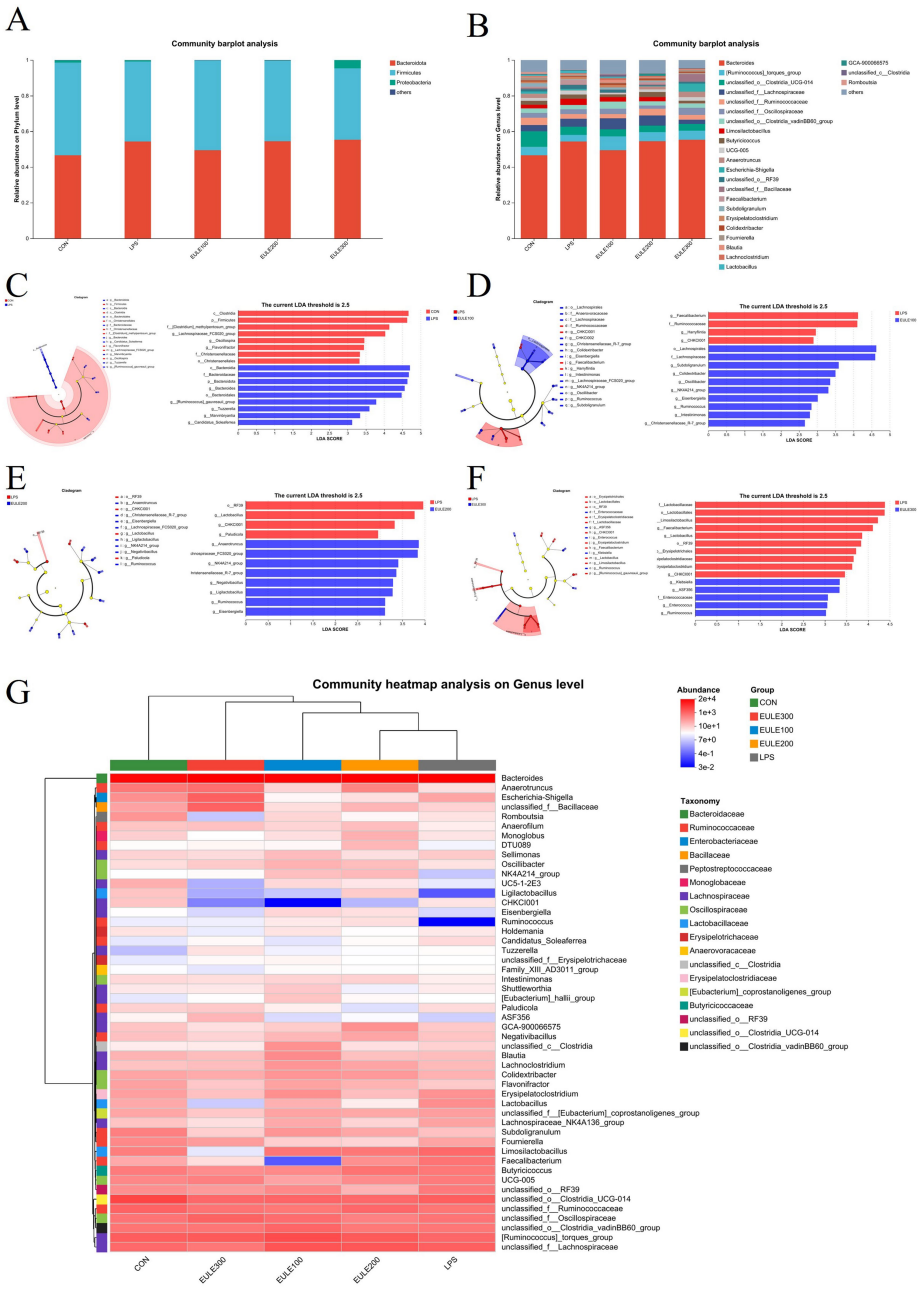
Effects of *Eucommia ulmoides* leaf extracts (EULE) on cecal microbiota composition in LPS-challenged broilers. **(A)** Microbial composition at the phylum level. **(B)** Microbial composition at the genus level. Difference between the cecal microbiota of CON and LPS groups of broilers **(C,D)** LPS and EULE100 groups, **(E)** LPS and EULE200 groups, and **(F)** LPS and EULE300 groups were determined by linear discriminant analysis effect size (LEfSe). **(G)** Clustering heatmap of the cecal microbial in each sample at the genus level. Red represents positive correlation and blue indicates negative correlation.

Taxonomic unit analysis revealed that the dominant phyla (Figure 4A; Table 5) of the five groups included Bacteroidetes (46.55, 54.30, 49.48, 54.45, 55.30%), Firmicutes (51.99, 44.81, 50.28, 45.14, 40.03%), and Proteobacteria (1.32, 0.76, 0.09, 0.18, 4.59%). The relative abundance of the ceca microbial community at phylum level was not affected by either LPS challenge or EULE supplementation (*p* > 0.05) (Table 5). At the genus level (Figure 4B; Table 6), the dominant microorganisms in the five groups were *Bacteroides* (46.55, 54.30, 49.48, 54.45, 55.30%), *Ruminococcus torques* (4.70, 3.70, 7.69, 5.13, 5.14%), *Clostridia_UCG-014* (8.83, 4.59, 3.94, 3.61, 3.68%), *Lachnospiraceae* (3.54, 4.53, 6.20, 5.74, 2.39%), *Ruminococcaceae* (4.01, 2.54, 2.37, 3.71, 2.68%), *Oscillospiraceae* (2.65, 2.67, 3.13, 1.90, 4.06%), *Clostridia_vadinBB60* (2.62, 2.41, 3.90, 2.35, 2.53%), *Limosilactobacillus* (2.03, 3.60, 2.55, 2.41, 0.04%), and *Butyricicoccus* (2.19, 2.33, 1.50, 2.78, 1.39%). The birds of LPS group had fewer *Faecalibacterium* compared to the other four treatment groups (*p* < 0.05) (Table 6). In addition to that, we can find that compared to the CON, LPS, and EULE200 groups, the number of *Erysipelatoclostridium* in the caecum of broilers in EULE100 group was significantly increased, while decreased significantly in the EULE300 group (*p* < 0.05) (Table 6).

**Table 5:** Effects of *Eucommia ulmoides* leaf extracts (EULE) on cecal microbiota composition in LPS-challenged broilers (the top five, at phylum level).

Microorganism	Dietary treatment					SEM	*p*-value
	CON	LPS	EULE100	EULE200	EULE300		
Bacteroidota	46.55%	54.30%	49.48%	54.45%	55.30%	0.017	0.471
Firmicutes	51.99%	44.82%	50.28%	45.14%	40.03%	0.021	0.428
Proteobacteria	1.32%	0.76%	0.09%	0.18%	4.59%	0.009	0.493
Actinobacteriota	0.08%	0.03%	0.09%	0.18%	0.02%	0.0003	0.579
Unclassified_d__Bacteria	0.06%	0.09%	0.07%	0.05%	0.06%	0.0001	0.777

CON, control group; LPS, challenged with LPS; EULE100, challenged with LPS and fed with the basal diet supplemented with 100 mg/kg EULE; EULE200, challenged with LPS and fed with the basal diet supplemented with 200 mg/kg EULE; EULE300, challenged with LPS and fed with the basal diet supplemented with 300 mg/kg EULE. LPS, Lipopolysaccharide; EULE, *Eucommia ulmoides* leaf extracts.

No shoulder label in the same row indicates no significant difference (*P* > 0.05).

**Table 6:** Effects of *Eucommia ulmoides* leaf extracts (EULE) on cecal microbiota composition in LPS-challenged broilers (at genus level).

Microorganism	Dietary treatmenta					SEM	*p*-value
	CON	LPS	EULE100	EULE200	EULE300		
*Bacteroides*	46.55%	54.30%	49.48%	54.45%	55.30%	0.017	0.471
[*Ruminococcus*]_*torques*_group	4.70%	3.70%	7.69%	5.13%	5.14%	0.006	0.275
Unclassified_o_*Clostridia*_UCG-014	8.83%	4.59%	3.94%	3.61%	3.68%	0.006	0.275
Unclassified_f_*Lachnospiraceae*	3.54%	4.53%	6.20%	5.74%	2.39%	0.005	0.111
Unclassified_f_*Ruminococcaceae*	4.01%	2.54%	2.37%	3.71%	2.68%	0.005	0.780
Unclassified_f_*Oscillospiraceae*	2.65%	2.67%	3.13%	1.90%	4.06%	0.004	0.558
Unclassified_o_*Clostridia*_vadinBB60_group	2.62%	2.41%	3.90%	2.35%	2.53%	0.005	0.876
*Limosilactobacillus*	2.03%	3.60%	2.55%	2.41%	0.04%	0.008	0.773
*Butyricicoccus*	2.19%	2.33%	1.50%	2.78%	1.39%	0.004	0.798
UCG-005	1.62%	2.18%	0.77%	1.45%	1.97%	0.004	0.829
*Anaerotruncus*	2.33%	0.16%	0.22%	1.70%	3.07%	0.006	0.545
*Escherichia*-*Shigella*	1.30%	0.75%	0.09%	0.17%	4.55%	0.009	0.503
Unclassified_o_RF39	1.29%	2.26%	1.53%	0.49%	0.98%	0.002	0.195
Unclassified_f_*Bacillaceae*	0.78%	0.23%	0.17%	0.50%	4.29%	0.007	0.357
*Faecalibacterium*	0.66%^ab^	0.00%^b^	2.67%^a^	1.28%^ab^	0.17%^ab^	0.003	0.034
*Subdoligranulum*	1.95%	0.36%	1.14%	0.70%	0.25%	0.004	0.604
*Erysipelatoclostridium*	0.87%^ab^	1.21%^ab^	1.30%^a^	0.40%^ab^	0.35%^b^	0.001	0.017
*Colidextribacter*	0.75%	0.53%	1.10%	0.99%	0.64%	0.001	0.295
*Fournierella*	1.53%	0.78%	0.25%	0.22%	0.94%	0.003	0.606
Unclassified_f_[Eubacterium]_*coprostanoligenes*_group	0.72%	0.90%	0.78%	0.68%	0.31%	0.001	0.626
*Blautia*	0.52%	0.39%	1.43%	0.37%	0.39%	0.002	0.613
*Lachnoclostridium*	0.34%	0.43%	1.14%	0.74%	0.39%	0.001	0.138
*Flavonifractor*	0.84%	0.29%	0.80%	0.50%	0.30%	0.001	0.419
*Lactobacillus*	0.66%	1.35%	0.55%	0.11%	0.02%	0.002	0.090
GCA-900066575	0.33%	0.29%	0.27%	1.26%	0.16%	0.002	0.606
*Lachnospiraceae*_NK4A136_group	0.33%	0.80%	0.66%	0.22%	0.16%	0.002	0.663
*Negativibacillus*	0.36%	0.34%	0.46%	0.74%	0.22%	0.001	0.117
Unclassified_c__*Clostridia*	0.12%	0.24%	1.27%	0.16%	0.13%	0.002	0.474
*Romboutsia*	1.13%	0.15%	0.21%	0.09%	0.02%	0.002	0.315
*Oscillibacter*	0.17%	0.15%	0.54%	0.40%	0.29%	0.001	0.334
Others	4.30%	2.90%	4.60%	4.79%	3.19%	0.003	0.276

Abbreviations: CON, control group; LPS, challenged with LPS; EULE100, challenged with LPS and fed with the basal diet supplemented with 100 mg/kg EULE; EULE200, challenged with LPS and fed with the basal diet supplemented with 200 mg/kg EULE; EULE300, challenged with LPS and fed with the basal diet supplemented with 300 mg/kg EULE. LPS = Lipopolysaccharide, EULE = Eucommia ulmoides leaf extracts.

^a,b^Values with different superscripts in the same row are significantly different (*P* < 0.05), no shoulder label in the same row indicates no significant difference (*P* > 0.05).

The LEfSe results showed that the ceca microbiota in the LPS group were enriched in Clostridia, Firmicutes, *Clostridium methylpentosum*, *Lachnospiraceae*, *Oscillospira*, *Flavonifractor*, Christensenellaceae, Christensenellales, while there were fewer Bacteroidia, Bacteroidaceae, Bacteroidota, *Bacteroides*, Bacteroidales, *Ruminococcus gauvreauii*, *Tuzzerella*, *Marvinbryantia*, *Candidatus soleaferrea* compared with the CON group (Figure 4C). In contrast to the LPS group, the microbiota in the EULE100 group were more abundant in Lachnospirales, Lachnospiraceae, *Subdoligranulum*, *Colidextribacter*, *Oscillibacter*, *NK4A214*, *Eisenbergiella*, *Ruminococcus*, *Intestinimonas*, *Christensenellaceae*, but showed fewer *Faecalibacterium*, Ruminococcaceae, *Harryflintia*, CHKC1001 (Figure 4D). In contrast to the LPS group, the microbiota in the EULE200 group were more abundant in *Anaerotruncus*, *Lachnospiraceae*, *NK4A214*, *Christensenellaceae*, *Negativibacillus*, *Ligilactobacillus*, *Ruminococcus*, *Eisenbergiella*, but showed fewer RF39, *Lactobacillus*, *CHKC1001*, *Paludicola* (Figure 4E). In contrast to the LPS group, the microbiota in the EULE300 group were more abundant in *CHKC1001*, *Klebsiella*, Enterococcaceae, *ASF356*, *Enterococcus*, *Ruminococcus*, but showed fewer Lactobacillaceae, Lactobacillales, *Limosilactobacillus*, *Faecalibacterium*, *Lactobacillus*, RF39, Erysipelotrichales, Erysipelatoclostridiaceae, *Erysipelatoclostridium* (Figure 4F). To further compare the variation in species composition and their species abundance distribution trends among groups, the top twenty genera of each sample in terms of relative abundance were grouped according to genus level and plotted on a heat map (Figure 4G). The CON group was significantly populated with *Bacteroides*, *RF39*, *Clostridia_UCG-014*, *Alistipes*, Ruminococcus_torques, and Lachnospiraceae. *Bacteroides*, *Ruminococcus_torques*, *Lachnospiraceae*, *Clostridia_UCG-014*, and *Limosilactobacillus* were greatly enriched in the LPS group. While *Bacteroides*, *Ruminococcus_torques*, *Lachnospiraceae*, *Clostridia_UCG-014*, and *Limosilactobacillus* were highly represented in EULE100 group. The EULE200 group were populated with *Bacteroides*, *Ruminococcus_torques*, *Lachnospiraceae*, *Clostridia_UCG-014* and *Ruminococcaceae.* The relative abundance of *Bacteroides*, *Ruminococcus_torques, Oscillospiraceae*, and *RF39* was increased in EULE300 group.

## 4 Discussion

Immune stress caused by LPS greatly impair the productive performance of chickens with grave economic consequences. Wang et al. (2023) reported that LPS injection significantly decreased (*p* < 0.05) egg laying rate, feed intake and feed efficiency. *Eucommia* bark and leaf extract exhibited noteworthy anti-stress and anti-oxidant activity in animal and human studies (Duan et al., 2024; Park et al., 2025). High dosage dietary (more than 1 g/kg dietary) CGA from *Eucommia ulmoides* extract has been shown to be able to modulate intestinal microbiota and antioxidant capacity, thereby improving broiler growth performance and intestinal health (Zhao et al., 2019; Liu H. et al., 2023; Hu et al., 2024). At present, it is not clear whether lower dose of EULE, such as 100, 200 or 300 mg/kg dietary supplementations, can promote the growth and health of broilers. In this study, the growth performance and relative organ weights of broilers were not affected by either LPS injection or EULE supplementation (*p* > 0.05). Based on relevant literature reports, it is speculated that if the supplement dose of EULE is increased or the feeding time is extended, it may be possible to achieve the effect of promoting the growth performance of broilers.

The cytokine level in serum is an important index to evaluate humoral immune response of poultry (Wu et al., 2018; Bai et al., 2022). IL-1β (one of proinflammatory cytokines) is a major coordinator of the immune response, which can stimulate immune cells to release a variety of cytokines to produce an immune response (Gu et al., 2020). In the present study, an increased IL-1β level was observed in the LPS group compared to the CON group, stimulation promoted the production of proinflammatory cytokines (IL-1β), while the difference is not significant. Dietary EULE significantly reduced IL-1β level in serum, which showed that EULE could counteract the inflammatory stress response induced by LPS injection. Many studies were in agreement with the results of this paper, which proved that EULE has anti-inflammatory activities (Huang et al., 2021; Duan et al., 2024). Supplemental EULE in the dietary exhibited significant anti-inflammatory effects by decreasing the level of IL-1β in heat-stressed broilers (Zhao et al., 2019). As is known to us all, proinflammatory cytokines TNF-*α*, IL-2, and IL-12 are excreted by Th1 cells, and anti-inflammatory cytokines IL-4, IL-5, and IL-10 secreted by Th2 cells (Wu et al., 2018). In this study, we found that the levels of IL-1β (Th1), IL-2 (Th1), IL-4 (Th2), and IL-6 (Th2) were increased after injection of LPS, indicating that the immune stress response was induced by the LPS stimulation. Compared with LPS group, the abnormal levels of IL-1β, IL-2, IL-4, and IL-6 were significantly altered by pre-adding EULE, especially supplemental 200 and 300 mg/kg EULE in feed exhibited significant anti-inflammatory effects by increasing the levels of anti-inflammatory cytokines (IL-4) and increasing the anti-inflammatory cytokines (IL-2, IL-6, IL-1β) levels, suggesting that EULE has significant anti-inflammatory and immune stress relieving potential. Based on those, we assume that the alteration of the cytokine level might be related to EULE’s anti-inflammatory potential through the regulation of Th1/Th2 cytokine secretion levels to repression of T-cell immune responses. However, the potential mechanisms of action should be verified by further animal studies. Immunoglobulin is a series of animal proteins with antibody activity, which mediates the body’s humoral immunity and is an important indicator of the body’s humoral immune function. Therefore, serum immunoglobulin levels reflect the level of immune function of the body to a certain extent (Liu et al., 2024). However, the excessive increase of immunoglobulin content is also a reflection of immune stress suffered by the body. Our research observed LPS injection caused an increase in serum IgA, IgG levels, compared with CON group. IgG and IgA, the two main kinds of immunoglobulins in birds, are clearly involved in the development of the serum immune response to LPS stimulation. Furthermore, in this paper, we also found that dietary 200 mg/kg EULE significantly alleviated the increase of IgA level, indicating that EULE has anti-inflammatory and immune stress effects. The improvement of serum IgG content by adding EULE did not reach a significant level, which may be due to the need for further research to explore the optimal dosage of EULE.

The stimulation of LPS not only induces inflammation but also leads to oxidative stress. Antioxidants derived from plant sources play a vital role in reducing oxidative processes and harmful effects of reactive oxygen species (ROS) (Gulcin, 2020). Chlorogenic acid, the main functional active substances of EULE, has been demonstrated that which can improve the antioxidant capacity of broilers by increasing the levels of antioxidant enzymes in many studies (Bai et al., 2022; Liu Y. et al., 2023; Liu et al., 2024). SOD, CAT, and GSH-Px are the most antioxidant enzymes in broilers, which can protect broiler body from oxidative damage caused by stress factors (Tang et al., 2019; Liu et al., 2022). In our study, the LPS challenge decreased the levels of CAT, SOD, GSH-Px and T-AOC, and increased the MDA level, which was consistent with a report by Zheng et al. (2016) and Dias et al. (2024). LPS injection causes the body to produce excess ROS, and the antioxidant enzyme system in the animal body can neutralize the excess ROS and maintain the balance of oxidation and antioxidant system in the body, thus eliminating oxidative damage (Zheng et al., 2016). The results of this study showed that compared with the CON group, the MDA level in LPS group was increased, and the SOD, T-AOC and CAT levels were decreased. The stress caused by LPS injection was inhibited by EULE dietary supplementation, and a dose of 300 mg/kg EULE in the diet had the best alleviating effect. These results indicated that EULE supplementation had a beneficial effect to protect tissues from lipid peroxidation as evidenced by reduced production of MDA and increased the level of SOD, T-AOC and CAT.

The intestinal microbiota worked together to maintain the homeostasis and health status of the intestinal internal environment of animals (Hu et al., 2024; Liu et al., 2024). More importantly, the chicken gut microbiota is a complex ecosystem for the host, responsible for converting food into nutrients and energy (Xu et al., 2016), and modulate the overall health and productiveness in poultry (Qin et al., 2018; Liu Y. et al., 2023). The diversity and composition of the intestinal microbiota are key elements to contribute the maintenance of a stable intestine microenvironment under stressed (Yi et al., 2023; Hu et al., 2024). The present study demonstrated that the alpha diversity was not affected by either LPS or dietary EULE, and the results of beta diversity analysis showed significant differences between groups. This suggest that even low dose for a short time of dietary supplemental EULE could change the ceca microbial community structure of LPS-induced stress broilers. In agreement with our results, Chen et al. (2021) found that supplementation with 300 and 600 mg/kg CGA in a basal diet of Hy-line brown pullets were not change the alpha diversity index of caca microbes in Hy-line brown pullets after heat stress. Our results of the PCA analysis indicted a distinction in the ceca microbial community composition among the five treatment groups. And notably, we observed that composition of ceca microbes under LPS-induced stress broilers improved with EULE intervention. Bacteroidetes was the most abundant phylum with the largest proportion in this study, followed by Firmicutes and proteobacteria, which consistent with the other report (Xu et al., 2016; Orso et al., 2021). These three phyla are also commonly observed in the gut environments of many birds (Wang et al., 2016; Chen et al., 2018; Shi et al., 2019). And the role of members of the three phyla in food digestion was frequently studied. For example, several species of Bacteroidetes metabolize polysaccharides and oligosaccharides to provide nutrition and vitamins (Zafar and Saier, 2021), Firmicutes members take part in insoluble fibre degradation (Berry, 2016), and species of Proteobacteria are related to the activity of cellulose (Reid et al., 2011; Shi et al., 2019). And researches reported that the changes in Firmicutes and Bacteroidetes phyla/species levels is frequently cited in the scientific literature as a hallmark of obesity (Indiani et al., 2018; Magne et al., 2020; Aragón-Vela et al., 2021). The members of Firmicutes such as *Clostridium leptum*, *Eubacterium hallii*, and *Lactobacillus* spp. indicated adipose tissue storage (Indiani et al., 2018). In this study, LEfSe analysis showed that the EULE supplementation enriched Bacteroidetes and *Bacteroides* in the ceca samples, although not at the level of difference. Xia et al. (2022) revealed that *Bacteroides* was negatively associated with IL-8 and IL-12 concentrations in heat-induced stress pigs, which could improve the immune function of the host. And Chen et al. (2018) reported that *Bacteroides* could maintain of other gut microbe balance, and might be improves unsaturated fatty acid synthesis. Bacteroidetes (the most dominant phylum) and *Bacteroides* (the most dominant genera), presented in all of the five treatment groups in the present study, and dietary EULE improved the numbers of Bacteroidetes and *Bacteroides*, which indicating that EULE supplementation was helpful for the broilers under LPS stressed. At genus level, LPS injection significantly decreased the number of *Faecalibacterium* compared with the CON group (*p* < 0.05), and which was not detected in the broilers of the LPS group. While dietary supplementation with 100 mg/kg EULE significantly increased the number of *Faecalibacterium* contrast to the LPS group (*p* < 0.05). Numerous studies have underscored that low levels of *Faecalibacterium* are correlated with inflammatory conditions (Miquel et al., 2013; Mohebali et al., 2023; Martín et al., 2023), and *Faecalibacterium* has the potential as a next generation probiotic or live biotherapeutic product (Martín et al., 2023). *Erysipelatoclostridium* is reported to enrich significantly in the radiation-induced intestinal injury (RIII) model (Li Z. et al., 2023). In this study, supplemental 300 mg/kg EULE in the feed exhibited reduced significantly the abundance of *Erysipelatoclostridium* in ceca. Interestingly, EULE treatment could shape the composition and diversity of ceca microbiota, increase the abundance of beneficial bacteria and decrease that of harmful genera. Together, the above experimental results strongly support how effective the addition of EULE to the diet of broiler chickens is in alleviating intestinal inflammation caused by immune stress and protecting the intestinal barrier from oxidative damage.

## 5 Conclusion

In summary, dietary supplementation with EULE could increase the feed intake of broiler breeders and alleviate their stress response to LPS stimulation to a certain extent. These findings imply that the possible roles of EULE are in preventing the inhibition of anti-oxidant function, immune response, and intestinal flora by LPS stimulation, and providing a new approach to counteract stress injury in broilers.

## Data Availability

The datasets presented in this study are publicly available. This data can be found here: https://www.ncbi.nlm.nih.gov, accession number PRJNA1332705.

## Footnotes

^1^ www.Majorbio.com
